# A rule-based energy management system for hybrid renewable energy sources with battery bank optimized by genetic algorithm optimization

**DOI:** 10.1038/s41598-024-54333-0

**Published:** 2024-02-28

**Authors:** Saif Jamal, Jagadeesh Pasupuleti, Janaka Ekanayake

**Affiliations:** 1 Department of Electrical and Electronics Engineering, College of Engineering, Universiti Tenaga Nasional, 43000 Kajang, Selangor Malaysia; 2 Institute of Sustainable Energy, Universiti Tenaga Nasional, 43000 Kajang, Selangor Malaysia; 3 Department of Electrical and Electronic Engineering, University of Peradeniya, Peradeniya, Sri Lanka

**Keywords:** Energy management system, Genetic algorithm, Renewable energy sources, Battery storage, Energy science and technology, Engineering

## Abstract

A Nanogrid (NG) model is described as a power distribution system that integrates Hybrid Renewable Energy Sources (HRESs) and Energy Storage Systems (ESSs) into the primary grid. However, this process is affected by several factors, like load variability, market pricing, and the intermittent nature of Wind Turbines (WTs) and Photovoltaic (PV) systems. Hence, other researchers in the past have used a few optimization-based processes to improve the development of Energy Management Systems (EMSs) and ESSs, which further enhanced the operational performance of NGs. It was seen that EMS acts as the distributed energy source in the NG setup and assists in power generation, usage, dissemination, and differential pricing. Hence this study employed the MATLAB Simulink software for modelling the grid-connected NG that included HRES; such as wind and PV; in addition to 3 Battery Storage Devices (BSDs) to design an effective EMS for the NG system and decrease its overall costs. For this purpose, a Rule-Based EMS (RB-EMS) that employs State Flow (SF) to guarantee a safe and reliable operating power flow to the NG has been developed. In addition to that, a Genetic Algorithm (GA)-based optimization system and Simulated Annealing optimization Algorithm (SAA) were proposed to determine an economical solution for decreasing the cost of the NG system depending on its operational constraints. Lastly, comparison about the cost between RB-EMS, GA and SAA has been presented. According to the simulation results, the proposed GA displayed an economical performance since it could achieve a 40% cost saving whereas the SAA system showed a 19.3% cost saving compared to the RB-EMS. It can be concluded from the findings that the GA-based optimization technique was very cost-effective displays many important features, like rapid convergence, simple design, and very few controlling factors.

## Introduction

A power distribution setup that can assimilate multiple distributed sources, like renewable energy sources (RESs), energy storage systems (ESSs), and non-RES, is known as a microgrid (MG) or nanogrid (NG). The power pyramid is what primarily distinguishes MG from NG. For example, NG is usually less complex and less powerful than MG. While MG can provide numerous entities, such as institutions of higher learning and medical and industrial facilities, with over tens of MWs, NG can provide a modest structure or a home with a power output exceeding tens of KWs^1^.

An energy management system (EMS) is described as a control mechanism that provides the necessary functionality and information to ensure that both the generation units and distribution setups produce electricity at the lowest possible operational costs in NG and MG applications^2,3^.

With the integration of RESs, NGs and MGs play a significant role in enhancing the electrical grid and moving away from sources of pollution. NG and MG systems use a variety of RESs, including solar photovoltaic (PV) and wind turbine (WT) systems. However, as PV and WT systems are intermittent in nature, an ESS consisting of supercapacitors or batteries can be used to sustain the power generated by such RES setups^4^. The output power levelling, the energy arbitrage, and the load-following functions of an ESS should be employed in conjunction with intermittent RESs^5^.

An NG is a unit that can be programmed to swiftly respond to the various load needs of local areas to increase the dependability of the supplied power, provide uninterrupted power supply, and decrease distribution losses. This can be accomplished using the NG’s power conversion system^6,7^. Most mid- and small-scale NGs derive their power from erratic and stochastic sources; such as WTs and PVs. However, load requirements and the grid tariffs change throughout the day. Due to these limitations and ambiguities, an NG is a complex system that requires intelligent control to ensure load demands can be met and it can be connected to the primary grid to keep its operational cost of electricity (COE) low^8^.

Multiple recent studies have examined how NG and MG systems function. A few optimization techniques have been used to identify the most effective operation schedules for various loading scenarios and objectives. One of the biggest issues that existing optimization methods face is the selection of the most cost-effective units to use^9^. Some studies have discussed several optimization techniques regarding the role of profit or cost in deciding the total power output of MG and NG applications^10,11^. For instance, in^12^ the authors have used recursive particle swarm optimization (rPSO) to increase reliance on RES and decrease reliance on traditional sources; like microturbines (MTs) and diesel generators (DGs). Although the suggested algorithm had higher efficacy than the Bat and GA algorithms, it took longer to address the issue than the Bat and GA algorithms. With fuel limits as a primary consideration, the authors in^13^ have suggested using the herd optimization algorithm (HOA) to schedule stand-alone NGs in the short run. The study's simulation-based findings indicated that the suggested approach was superior to previous methods.

In^14^, the researchers developed two methodologies; namely, a heuristic that utilizes the SF method and an optimization algorithm based on linear programming (LP); to reduce the cost of grid-connected MGs composed of a BSD and a PV array. The projected cost and loading conditions were considered during the optimization procedure. The simulation outcomes showed that the suggested optimization approach reduced operational costs by about 19%.

In^15^, the authors created a grey wolf optimisation (GWO) method to reduce the operational costs of grid-connected MGs; such as WTs, PV arrays, MTs, BSDs, and fuel cells (FCs). The operating cost solely applied to MTs, and FCs consist of operation and maintenance (O&M), fuel, and start-up costs, and shut-down expenses. Notably, the start-up and shut-down costs for WTs, PVs, and BSDs were zero. Furthermore, the ideal BSD size was also considered to lower MG operating expenses. The simulation data indicated that the devised optimization technique was more effective than differential evolution (DE) and particle swarm optimisation (PSO).

Another study proposed a grid-connected MG EMS with solar and BSD to reduce an MG's COE. Heuristic and optimization strategies for clear and foggy days were both considered. The simulation outcomes revealed that the optimization approach decreased the COE by 6.6% on clear days and 13.7% on overcast days than heuristics^16^.

A teacher-learning-based optimization (TLBO) was used to overcome the non-linear energy management issues and decrease the operating cost of a grid-connected MG composed of a WT, a PV, a FC, a MT, and a BSD. The simulation findings showed that the suggested method could perform a global search, resulting in quick and acceptable convergence, while the experimental findings demonstrated its superiority and viability over other prominent methods^17^.

In^18^, the researchers created an RB-EMS optimized using a grasshopper optimisation algorithm (GOA) to plan the capacity of independent MGs made up of a WT, a PV array, a DG, and a BSD. The RB-EMS was used to increase the use of RESs and to guarantee power flow inside the MG system. The effectiveness of the planned GOA-integrated RB-EMS were also determined and validated. The simulation outcomes demonstrated that the suggested RB-EMS was a cleaner power production mechanism as it reduced fuel consumption, CO_2_ emission, and COE by 92.4%, 92.3%, and 79.8%, respectively, than a traditional DG. A comparison of the algorithms indicated that the GOA-integrated RB-EMS yielded the best results as its COE (USD0.3656/kWh) was lower than that of a cuckoo search algorithm (CSA)-integrated RB-EMS (USD0.3662/kWh).

In^19^, the authors presented an improved RB-EMS to guarantee the dependable operation of a freestanding MG comprising a WT, a PV, a DG, and a BSD. The best RB-EMS was improved based on comparisons of the RB-EMS performances indicated by earlier efforts. The simulation outcomes demonstrated that, in comparison to existing RB-EMS, the suggested technique resulted in fewer emissions, costs, operational outlays, and power losses.

In^20^, the researchers proposed an EMS based on stochastic model predictive control (SMPC) for grid-connected MGs with a WT, a PV, a DG, a FC, and a BSD. A mixed integer quadratic programming model was devised to reduce the operating cost. A thorough comparison of the simulations indicated that the suggested algorithm yielded lower operating cost than other state-of-the-art approaches.

In^21^, the researchers proposed an ideal EMS, that consisted of a FC, a PV, and a BSD, for a grid-connected NG to decrease its operational cost and determine the ideal BSD size. Linear programming (LP) was used for objective functions with non-integer variables to discover the best solutions. The best solution for objective functions with integer variables was also discovered using mixed-integer linear programming (MILP). The findings showed that the suggested hourly-based optimal operation mode reduced daytime operating costs by 17.8 to 94.5%.

A hierarchical control architecture is necessary to regulate NGs. This hierarchy has three control levels; namely, primary, secondary, and tertiary. Local voltage, frequency, and current are controlled at the primary control level or power management system (PMS) level^1^.

The EMS or secondary control level manages and regulates the power flow between the generation sources, the primary grid, and the internal load needs of the NG system^1^. The tertiary control level deals with the connection between the network and the NG^22^. This paper will focus on the EMS level; namely, the second level of NG control.

**Figure 1:**
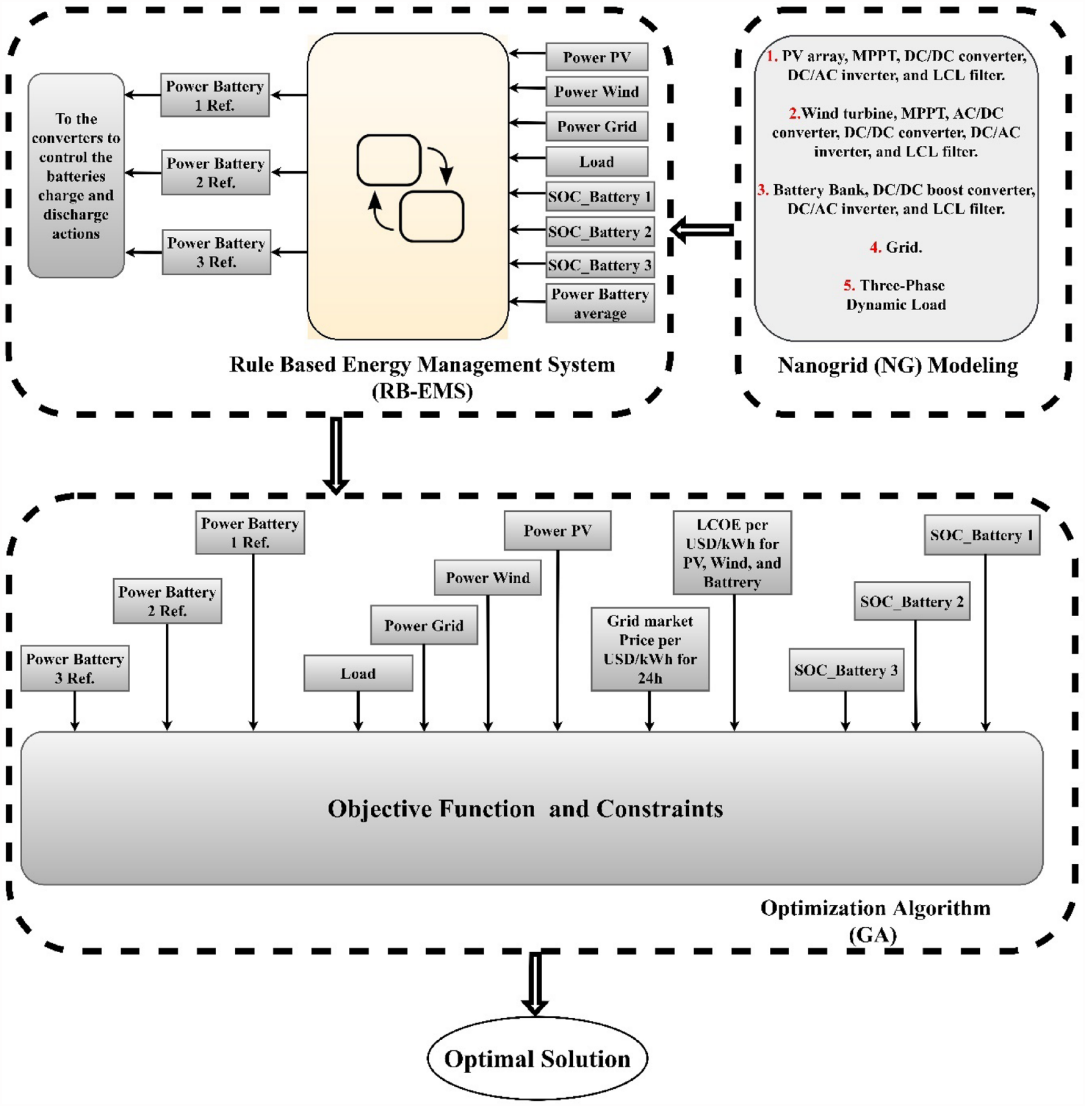
RB-EMS and GA based on grid connected NG.

The research objectives were split into three steps. The first step was to model the grid-connected NG using MATLAB Simulink, which included a WT and PV hybrid RES dual active bridge (DAB) DC/DC converters, a BSD, LCL filters, DC/AC inverters, and the variable load for one day. In the second step, a rule-based EMS using an SF approach for an NG system to ensure power flow and a safe and dependable operation was recommended. The third stage involves applying an optimization technique based on a genetic algorithm (GA) for an NG system to find the most economical solution for distributed generation units. To showcase the effectiveness of the recommended algorithm, the performance of GA is compared to that of the Simulated Annealing optimization algorithm (SAA). Figure 1 depicts the frameworks of all the procedures used to determine the most advantageous and cost-effective solutions for grid connected NGs. The primary contributions of this paper are summarised below:

1. Designed the NG-connected modelled grid using the MATLAB Simulink software and proposed RB-EMS for guaranteeing a safe and reliable operating power flow in the NG system.
2. Analysis of the single-objective optimization issue after decreasing the overall operational costs.
3. GA has been introduced for resolving the single-objective optimization issue.
4. GA displays many important features, like rapid convergence, simple design, and very few controlling factors.
5. The simulation results were compared with the SAA optimization findings.
6. Finally, the effectiveness and robustness of the proposed GA in the single-objective optimization setting were validated.

The paper is structured as follows: Section “Introduction” introduces MG and NG energy management. Section “Nanogrid (NG) system explanation” gives a comprehensive description of the NG system with RB-EMS. Section “Formulation of optimization problem” describes the optimization problem, objective function, limitations, and GA execution. Section “Simulation results and discussions” presents the outcomes and discussion. The conclusions are presented in Section “Conclusions”.

## Nanogrid (NG) system explanation

**Figure 2:**
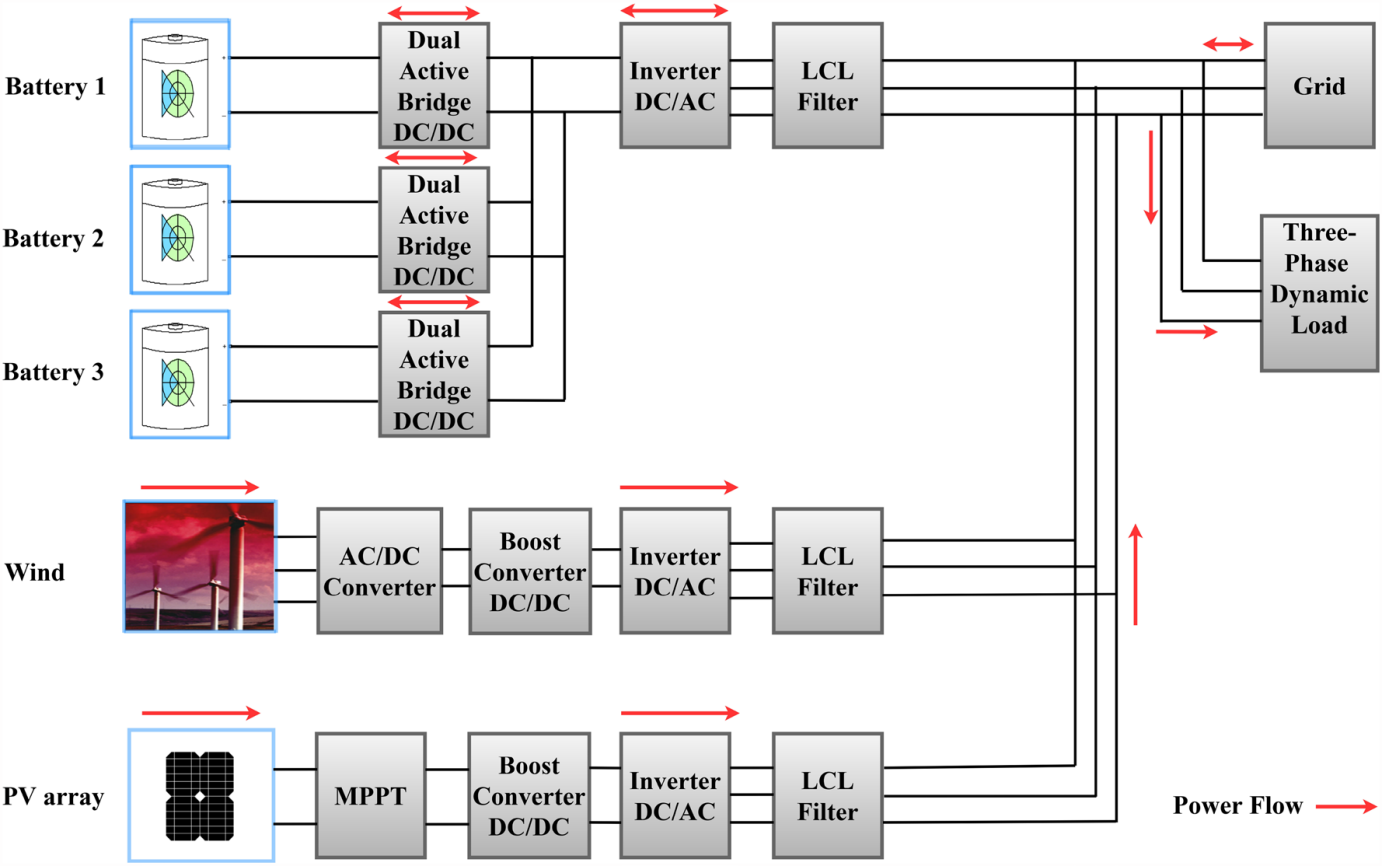
Nanogrid (NG) modeling.

As seen in Fig. 2, the grid-connected NG comprised a PV array, a BSD, a WT system, a DC/DC boost converter, DAB converters, LCL filters, and DC/AC inverters, along with three-phase loads modelled in MATLAB Simulink software coupled with local controllers.

Maximum power point tracking (MPPT) extracted energy from the PV array while the BSD created energy balance in the NG structure. The WT system, which was a critical component, was designed using a permanent magnet synchronous generator (PMSG).

### Photovoltaic (PV)

The array, which was simulated in MATLAB, comprised 14 series-connected PV panels and four parallel connections. The parameters of the PV panels were set as maximum current output (I_M_) = 8.18 A, maximum voltage output (V_M_) = 36.7 V, short circuit current (I_SC_) = 8.68 A, open circuit voltage (V_OC_) = 45.3 V, maximum power (P_M_) = 300 W, and number of cells = 72.

**Figure 3:**
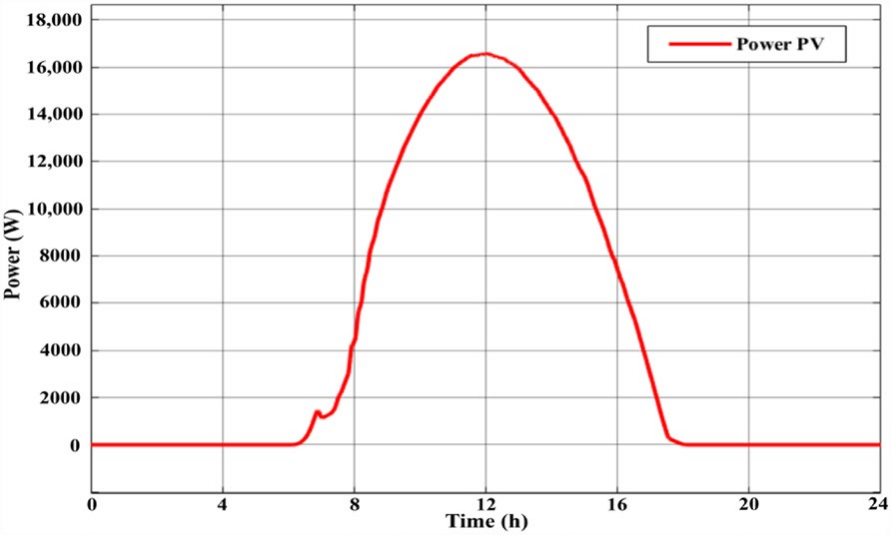
The PV array power curve for one day.

**Figure 4:**
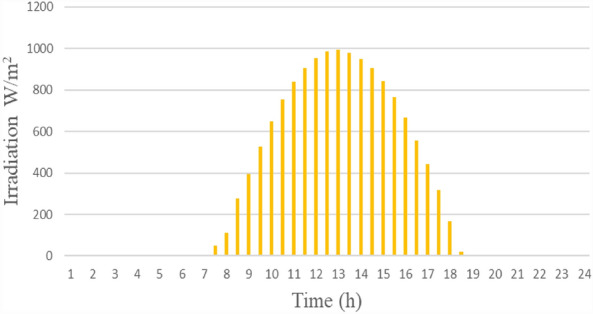
The PV irradiation data for one day.

Figure 3 depicts the power characteristics of the PV system using Malaysian irradiation data for 24 h at 25 °C. The peak power production was 16.7 kW. Figure 4 depicts the single-day irradiation information. The perturb and observe (P&O) technique was used to drive the MPPT to extract power from the PV array^23^. The algorithm computed the duty cycle required to drive the DC/DC boost converter to extract optimum power from the PV system.

The equation below was used to compute the highest current output of the PV array

1 $${{\text{P}}}_{{\text{PV}}\_{\text{MAX}}}={{\text{V}}}_{{\text{M}}}{{\text{I}}}_{{\text{M}}}$$

where, V_M_ is the maximum voltage output of the PV array and I_M_ is its highest current output.

### Wind turbine system (WTS)

The WT system comprised a PMSG-based WT, a DC/DC boost converter, a DC converter, a DC/AC inverter, and a controller. The wind-based power computation is, subsequently, discussed.

2 $${{\text{P}}}_{{\text{M}}\_{\text{PU}}}={{\text{K}}}_{{\text{P}}}{{\text{C}}}_{{\text{P}}\_{\text{PU}}}{{\text{V}}}_{{\text{WIND}}\_{\text{PU}}}$$

where, P_M_PU_ is the nominal power in per unit (PU) for the specific ρ (air density in kg/m3) and A (swept area in m^2^) values; K_P_ is the power gain (K_P_ was ≤ 1), C_P___PU_ is the performance coefficient in PU of the maximum c_p; and_ V_WIND_PU_ is the wind speed in PU of the base wind speed. The base wind speed is the mean value of the expected wind speed in m/s^24^.

**Figure 5:**
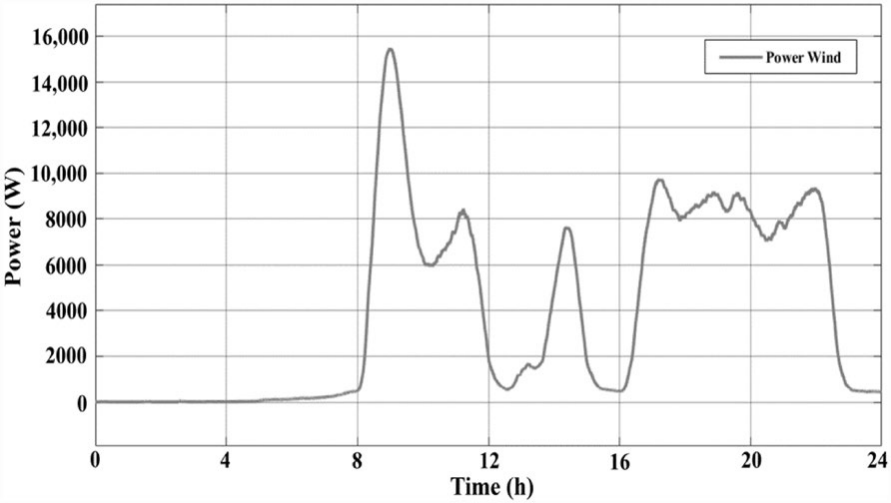
The power curve of wind for one day.

**Figure 6:**
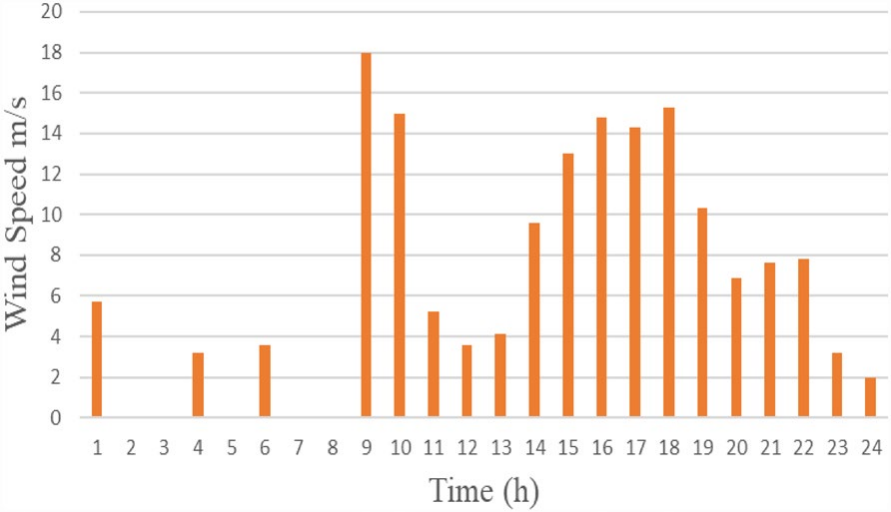
The wind speed data for one day.

A variable-speed WT system was employed for 24 h. Figure 5 indicates single-day power characteristics with a 15-kW peak. Moreover, Fig. 6 indicates single-day wind speed information.

### Battery storage devices

It was critical to connect a BSD to the grid-linked system due to the uncertain power generation of PV and WT sources. The BSD comprised three lithium-ion batteries that had several desirable characteristics; such as high energy density, high performance, superior life cycle (1000 cycles), and low power duration (within 1 h)^25^.

The BSD charging and discharging states depended on the state of charge (SOC), the WT’s available power, and the PV’s available power. The SOC thresholds determine the amount of energy drawn from the BSD.

3 $${\text{SOC}}\_{\text{MIN}}\le {\text{SOC}}\le {\text{SOC}}\_{\text{MAX}}$$

where, SOC_MIN and SOC_MAX are the lower and the upper limits of the SOC of the BSD, respectively. Table 1 provides the parameters of the BSD.

**Table 1:** The battery storage parameters**.**

Parameters	Values
Operating voltage range	42–54 V
Nominal voltage	48 V
Rated capacity	100 Ah
Initial SOC	40%
SOC_MAX	80%
SOC_MIN	20%

### DC/DC converters

As a DC/DC boost converter is a very efficient transformer-less device that steps up the input voltage, it is frequently used in WT and PV installations^26^. Table 2 lists the DC/DC boost converter parameters of the PV and WT. A DAB converter, which was initially recommended by^27^, was also discussed in^5^. A DAB may be considered a bidirectional DC/DC converter comprising galvanic isolation-based on DAB, a high-frequency transformer, and leakage inductance. The transformer provides galvanic isolation with very high conversion. Hence, a DAB is used if the input and output voltages vary significantly. The converter was placed after the BSD. Table 3 lists the DAB converter aspects of the BSD.

**Table 2:** The DC/DC boost converter parameters**.**

Parameters	Values of PV side converter	Values of wind side converter
Converter switching frequency (fsw_boost)	25 kHz	25 kHz
Inductance (L)	1.53 × 10^3^ H	1.53 × 10^3^ H
Input capacitor (Cin)	100 × 10^6^ F	100 × 10^6^ F
Output capacitor (Cout)	1 × 10^3^ F	1 × 10^3^ F

**Table 3:** The DC/DC DAB converter parameters**.**

Parameters	Values
Input capacitance (C_in_)	2000 × 10^6^ F
Output capacitance (Co)	2000 × 10^6^ F
Leakage inductance (L)	6 × 10^6^ H
Switching frequency (Fs)	25 kHz
Input voltage (Vin)	48 V
Output voltage (Vo)	800 V
Maximum duty cycle (d_MAX_)	0.35
Turn ratio of the transformer (n)	5

### DC/AC inverters and LCL filters

MG and NG setups critically rely on DC/AC inverters to transform the DC power from PV arrays, WTs, or BSDs to AC power. A more comprehensive analysis was presented in^28^.

LCL filters help eliminate unnecessary harmonics. The technique specified by^27^ was employed to power a converter-specific LCL filter design. Table 4 lists the LCL filters and the DC/AC inverter parameters.

**Table 4:** The parameters of DC/AC inverter LCL filters.

Parameters	Values of PV inverter and LCL filter	Values of WT inverter and LCL filter	Values of batteries inverter and LCL filter
Rated power (Pn)	16.7 kW	15.0 kW	15.0 kW
DC voltage (${{\text{V}}}_{{\text{DC}}}$)	800 V	800 V	800 V
Grid voltage (Vg)	400 V	400 V	400 V
Grid frequency (Fg)	50HZ	50HZ	50HZ
Switching frequency (Fsw)	10kHZ	10kHZ	10kHZ
Filter capacitor (C)	32.22 µF	32.22 µF	40 µF
Inverter-side inductor (L1)	3 mH	3 mH	2.55 mH
Grid-side inductor (L2)	0.046 mH	0.046 mH	0.038 mH

### Rule-based energy management system (RB-EMS) using state flow (SF)

The SF technique was modified to suit the RB-EMS approach. This technique comprised an event-specific modelling toolbox in MATLAB to facilitate the logic modelling of the dynamic regulation of the RB-EMS. The power flow regulation is based on the energy availability data of the BSD, WT, PV, and energy demand. It provides control commands to power conversion systems that include the converters and inverters typically used to integrate NG systems.

The RB-EMS was the primary instrument that controlled and regulated the commands in the NG setup and the workings of all the inverters, controllers, and converters while the MPPT drove the DC/DC boost converters connected to the PV and WT systems. The DAB-only converters of the BSD controlled its discharging or charging states to provide a steady DC bus voltage. It was critical to balance the NG power for various power levels from the BSD, the PV, the WT, the grid, and the energy demand. The power balance expression is specified below.

4 $${{\text{P}}}_{{\text{PV}}}+{{\text{P}}}_{{\text{WIND}}}+{{\text{P}}}_{{\text{BATTERIES}}}+{{\text{P}}}_{{\text{GRID}}}={{\text{P}}}_{{\text{LOAD}}}$$

**Figure 7:**
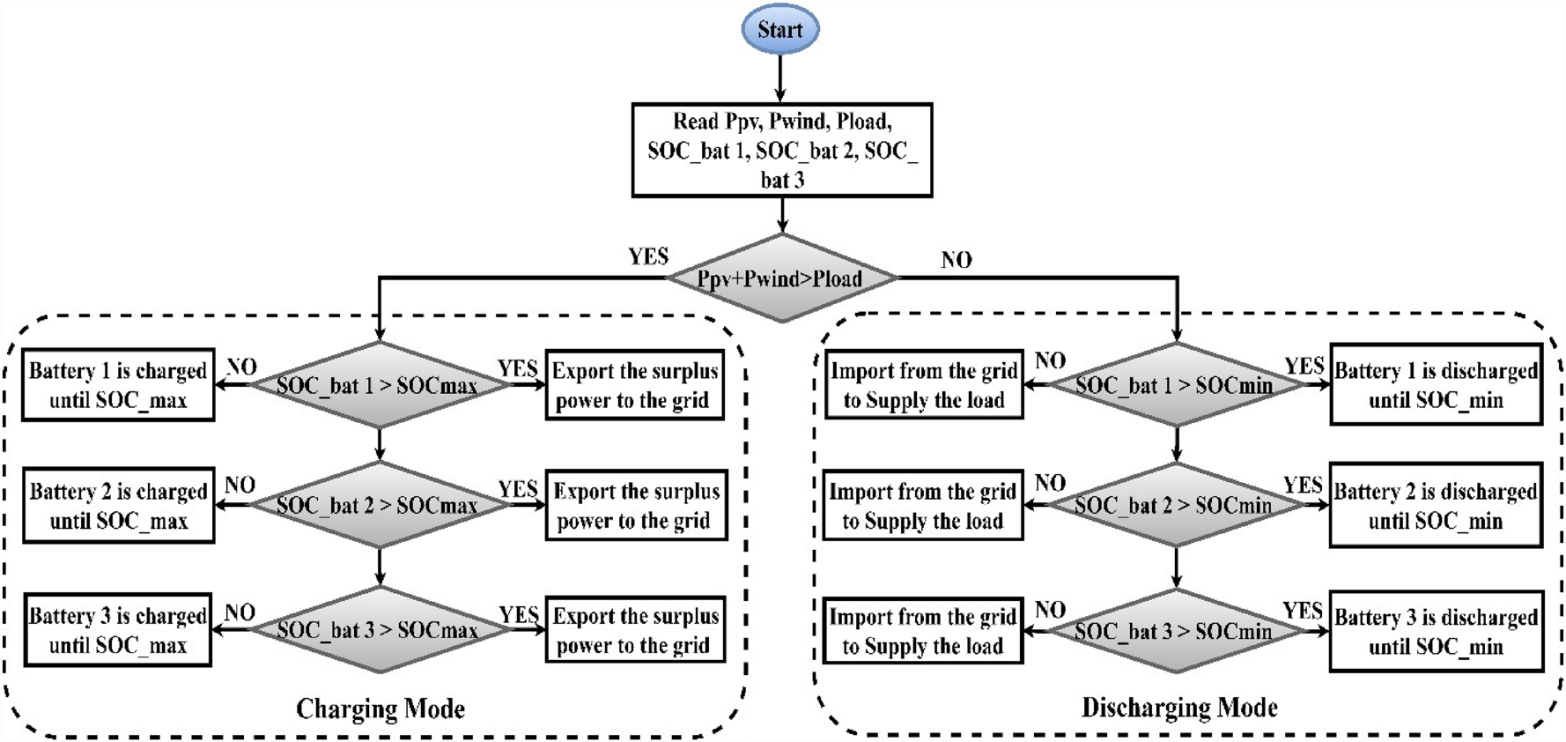
Rule based energy management system (RB-EMS).

Figure 7 presents a flowchart of the various operation modes of the RB-EMS applied in the SF.

The EMS used different modes based on two criteria; namely, power production from the WT and the PV and the SOC of the BSD.

If the power demand exceeded the average power generated by the WT and PV systems, the BSD balanced the deficit until the SOC approached SOC_MIN. The three BSDs discharged equally to balance the power. For instance, if the BSD was powering a 3-kW load, each BSD was loaded at 1 kW. The grid was the last source used to power the balance, which was when the BSDs reached SOC_MIN. The more desirable scenario was the average power generated by the WT and PV system exceeding the load demand. The PV and WT systems powered the load demand and charged the BSD to SOC_MAX. When the SOC_MAX limit was reached, the excess energy was exported to the grid.

## Formulation of optimization problem

This section specifies the optimization problem of this present study. The overall operating cost of the NG was the objective function. The decision variables comprised DG power generation and the grid to provide optimal power production points for every source. ON/OFF states were used to optimize the overall operating cost of the NG and fulfil the restrictions concerning power generation levels, load balance, and BSD charging/discharging.

### Objective functions

As reducing the total cost is critical to optimizing NG operations, various objective functions for reducing the cost of NG and MG have been presented in^29,30^. The suggested expressions considered the levelized cost of energy (LCOE), which comprises the capital investment and O&M capital corresponding to every source comprising the NG setup. The primary target of the recommended objective functions was to economically fulfil load demands throughout the day. The following function was suggested to decrease the total operating cost of the NG system:

5 $${\text{Minf}}({\text{x}})={\sum }_{{\text{t}}=1}^{{\text{T}}}\left\{{\sum }_{{\text{i}}=1}^{{{\text{N}}}_{{\text{g}}}}\left[{\mathrm{ u}}_{{\text{i}}}({\text{t}}){{\text{P}}}_{{\text{gi}}}({\text{t}}){{\text{B}}}_{{\text{gi}}}({\text{t}})\right]+{\sum }_{{\text{j}}=1}^{{{\text{N}}}_{{\text{Bat}}}}\left[{{\text{u}}}_{{\text{j}}}({\text{t}}){{\text{P}}}_{{\text{Batj}}}({\text{t}}){{\text{B}}}_{{\text{Batj}}}({\text{t}})\right]+({{\text{P}}}_{{\text{Grid}}}\left({\text{t}}\right){{\text{B}}}_{{\text{Grid}}}\left({\text{t}}\right))]\right\}$$

6 $${{\text{P}}}_{{\text{gi}}}({\text{t}})=\left[{{\text{P}}}_{{\text{PV}}}({\text{t}}),{{\text{P}}}_{{\text{WIND}}}({\text{t}}),\dots \dots \dots {{\text{P}}}_{{{\text{N}}}_{{\text{g}}}}({\text{t}})]\right.$$

7 $${{\text{P}}}_{{\text{PV}}}({\text{t}})=\left[{{\text{P}}}_{{\text{PV}}}(1),{{\text{P}}}_{{\text{PV}}}(2),{{\text{P}}}_{{\text{PV}}}(3),\dots \dots \dots {{\text{P}}}_{{\text{PV}}}({\text{T}})]\right.$$

8 $${{\text{P}}}_{{\text{WIND}}}({\text{t}})=\left[{{\text{P}}}_{{\text{WIND}}}(1),{{\text{P}}}_{{\text{WIND}}}(2),{{\text{P}}}_{{\text{WIND}}}(3),\dots \dots \dots {{\text{P}}}_{{\text{WIND}}}({\text{T}})]\right.$$

9 $${{\text{P}}}_{{\text{Batj}}}({\text{t}})=\left[{{\text{P}}}_{\mathrm{Bat }1}({\text{t}}),{{\text{P}}}_{\mathrm{Bat }2}({\text{t}}),{{\text{P}}}_{\mathrm{Bat }3}({\text{t}}),\dots \dots \dots {{\text{P}}}_{{{\text{N}}}_{{\text{Bat}}}}({\text{t}})]\right.$$

10 $${{\text{P}}}_{\mathrm{Bat }1}({\text{t}})=\left[{{\text{P}}}_{\mathrm{Bat }1}(1),{{\text{P}}}_{\mathrm{Bat }1}(2),{{\text{P}}}_{\mathrm{Bat }1}(3),\dots \dots \dots {{\text{P}}}_{\mathrm{Bat }1}({\text{T}})]\right.$$

11 $${{\text{P}}}_{\mathrm{Bat }2}({\text{t}})=\left[{{\text{P}}}_{\mathrm{Bat }2}(1),{{\text{P}}}_{\mathrm{Bat }2}(2),{{\text{P}}}_{\mathrm{Bat }2}(3),\dots \dots \dots {{\text{P}}}_{\mathrm{Bat }2}({\text{T}})]\right.$$

12 $${{\text{P}}}_{\mathrm{Bat }3}({\text{t}})=\left[{{\text{P}}}_{\mathrm{Bat }3}(1),{{\text{P}}}_{\mathrm{Bat }3}(2),{{\text{P}}}_{\mathrm{Bat }3}(3),\dots \dots \dots {{\text{P}}}_{\mathrm{Bat }3}({\text{T}})]\right.$$

13 $${{\text{P}}}_{{\text{Grid}}}({\text{t}})=\left[{{\text{P}}}_{{\text{Grid}}}(1),{{\text{P}}}_{{\text{Grid}}}(2),{{\text{P}}}_{{\text{Grid}}}(3),\dots \dots \dots {{\text{P}}}_{{\text{Grid}}}({\text{T}})]\right.$$

where, T is the total time of the study in hours (h); ${{\text{N}}}_{{\text{g}}}$ and ${{\text{N}}}_{{\text{Bat}}}$ are the energy generation units and BSD, respectively; Ui (t) is the status of the generation and BSD units at time t, either in ON or OFF mode; ${{\text{P}}}_{{\text{gi}}}({\text{t}})$ and ${{\text{P}}}_{{\text{Batj}}}({\text{t}})$ are the amount of power output by the generation units and BSD at time t; ${{\text{B}}}_{{\text{gi}}}({\text{t}})$ and ${{\text{B}}}_{{\text{Batj}}}({\text{t}})$ are the energy price offered for each generated unit and BSD at time t; and ${{\text{P}}}_{{\text{Grid}}}({\text{t}})$ and ${{\text{B}}}_{{\text{Grid}}}\left({\text{t}}\right)$ are the volume of power exchanged and the grid market price at time t.

### Constraints and limitations

Three constraints; namely, the load generation balance, the power limit of the generating units, and the charging and discharging of the BSD; were considered.

#### Load generation balance

As load demands must be fulfilled, regardless of the conditions, the NG should be able to adequately fulfil the load demand. This case can be expressed as follows:

14 $${\sum }_{{\text{i}}=1}^{{{\text{N}}}_{{\text{g}}}}{{\text{P}}}_{{\text{gi}}}\left({\text{t}}\right) \, + \, {\sum }_{{\text{j}}=1}^{{{\text{N}}}_{{\text{Bat}}}} {{\text{P}}}_{{\text{Batj}}}\left({\text{t}}\right) + {{\text{P}}}_{{\text{Grid}}}\left({\text{t}}\right) = \, {\sum }_{{\text{k}}=1}^{{{\text{N}}}_{{\text{k}}}}{{\text{PL}}}_{{\text{k}}}\left({\text{t}}\right)$$

where, ${{\text{PL}}}_{{\text{k}}}\left({\text{t}}\right)$ is the load demanded at hour t.

#### Power limit of units

The amount of power that the WT, PV, and BSDs can provide are subject to the following limitations:

15 $$\begin{aligned} &{{\text{P}}}_{{\text{gi}}}\mathrm{ MIN}\left({\text{t}}\right) \le {{\text{P}}}_{{\text{gi}}}\left({\text{t}}\right) \le {{\text{P}}}_{{\text{gi}}}\mathrm{ MAX}\left({\text{t}}\right) \\ & {{\text{P}}}_{{\text{Batj}}}\mathrm{ MIN}\left({\text{t}}\right) \le {{\text{P}}}_{{\text{Batj}}}\left({\text{t}}\right) \le {{\text{P}}}_{{\text{Batj}}}\mathrm{ MAX}\left({\text{t}}\right) \end{aligned}$$

where, MAX and MIN are the maximum and minimum boundaries of the variable, respectively.

#### Battery storage charging and discharging constraint

The following expressions specify the charge and discharge limits of the BSD^31^:

16 $${{\text{W}}}_{{\text{Bat}},{\text{t}}} = {{\text{W}}}_{{\text{Bat}},{\text{t}}-1} + {\upeta }_{{\text{charge}}}{{\text{P}}}_{{\text{charge}}}\mathrm{\Delta t }- \frac{1}{{\upeta }_{{\text{discharge}}}} {{\text{P}}}_{{\text{discharge}}}\mathrm{\Delta t}$$

17 $$\left\{\begin{array}{c}{{\text{W}}}_{{\text{Bat}},{\text{MIN}}} \le {{\text{W}}}_{{\text{Bat}},{\text{t}}} \le {{\text{W}}}_{{\text{Bat}},{\text{MAX}}}\\ \, \, {{\text{P}}}_{{\text{charge}},\mathrm{t }}\le {{\text{P}}}_{{\text{charge}},\mathrm{ MAX}}; \, \, {{\text{P}}}_{{\text{discharge}},\mathrm{ t}} \le {{\text{P}}}_{{\text{discharge}},\mathrm{ MAX}}\end{array}\right\}$$

where, W_Bat_(t) and W_Bat_ (t − 1) are the volume of energy stored in the BSD at times t and t − 1, respectively; ${{\text{W}}}_{{\text{Bat}},{\text{MIN}}}$ and ${{\text{W}}}_{{\text{Bat}},{\text{MAX}}}$ are the lowest and highest amount of energy stored in the BSD, respectively; ${{\text{P}}}_{{\text{charge}},\mathrm{ MAX}}$ and ${{\text{P}}}_{{\text{discharge}},\mathrm{ MAX}}$ are the maximum charging and discharging powers of the BSD, respectively; ${{\text{P}}}_{{\text{charge}}}$ and ${{\text{P}}}_{{\text{discharge}}}$ are the maximum charging and discharging powers of the BSD over a given timeframe, respectively; and ${\upeta }_{{\text{charge}}}$ and ${\upeta }_{{\text{discharge}}}$ are the efficacy of the BSD in the charging and discharging modes, respectively.

### System variables

This section provides a comprehensive overview of aspects of the system; such as the energy production of the WT and PV, the load characteristics, and the grid tariffs of the NG setup. All the assumptions discussed in this section were based on real-world data.

#### PV array and wind power availability

As wind velocity and irradiation levels determine the power production of WT and PV systems, they were modelled based on the Malaysian climate. Figures 3 and 5 provide the hourly predicted PV and wind power levels throughout the day, respectively.

#### Load profile

**Figure 8:**
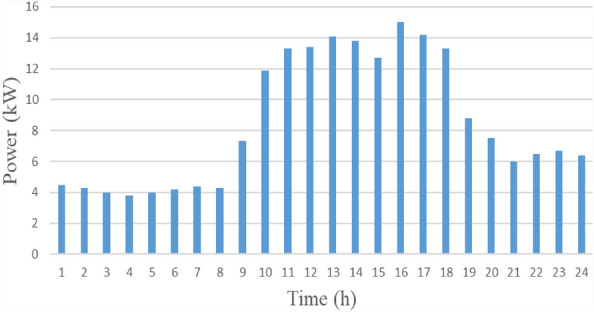
Load profile for one day.

Numerous factors; such as geography, hourly time of day, and climate change; affect load demand. Figure 8 provides the random commercial load profile for Malaysia, with time-specific changes in load demand indicated^5^.

#### Malaysian grid tariff

**Figure 9:**
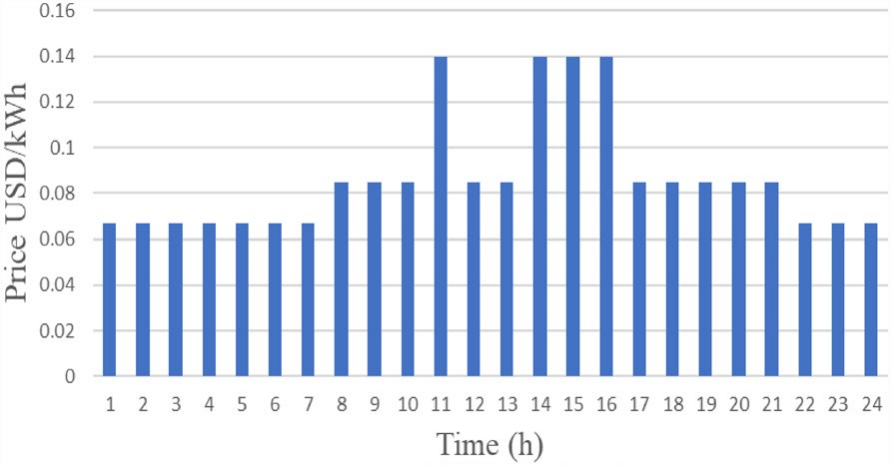
Malaysian power price for one day.

The primary grid market price indicates changes in demand while supply pricing facilitates higher production during peak demand and lesser production during lower demand. Figure 9 provides the 24 h electricity price (USD/kWh) profile of the Malaysian national grid^32^. The indicated price reflects traditional commercial requirements and is based on Tenaga Nasional Berhad (TNB). The daily commercial electricity price can be split into three intervals; namely, 4 h of peak demand at 11, 14, 15, and 16; 10 h of mid-peak demand at 8 to 11, 12 to 14, and 17 to 22; and 10 h of off-peak demand from 22 to 8.

### System parameters

This section specifies the source-specific power levels, including the LCOE of a DG when it is in operation and the true cost of using a BSD.

Wind turbines (WTs), PV arrays, and BSDs have upper and lower power generation constraints during operation. The International Renewable Energy Agency (IRENA) specifies the LCOE per kWh standards for BSD and RES systems. As the LCOE depends on the country, the data used in this present study pertained to Malaysia. The LCOE typically comprises the installation and O&M cost of a power source. Furthermore, the true cost of using a BSD (USD/kWh) may be computed by amortising its cost (USD135/kWh per IRENA standards) over its usable life. The usable life may be determined by multiplying the cycle life (3000 cycles) and the rated energy content of the BSD (4.8 kWh). Table 5 lists the actual LCOE of PV arrays, WTs, and BSDs based on their usable life as well as upper and lower limits for BSDs and RESs.

**Table 5:** LCOE and technical coefficient of the DGs sources.

Unit	LCOE (USD/kWh)	P_MIN_ (kW)	P_MAX_ (kW)
PV	0.078	0	16.7
Wind	0.039	0	15
Battery	0.045	− 4.8	4.8

### Genetic algorithm (GA)

This section discusses the optimization algorithm for the power generated by the NG system. The GA was employed to assess the optimization issues of NG operation^33^. The GA can be defined as a stochastic global search algorithm that facilitates the simulation of a metaphor of a natural biological evolution^34,35^.

Optimizing using GA offers several benefits over other algorithms, including optimizing with continuous or discrete variables and not requiring derivative information. The GA can also deal with non-linearity issues, whether as constraints or objective functions^36^. It can also handle numerous variables and function with experimental data, analytical functions, or numerically generated data.

Like all the optimization algorithms, the GA needs the specification of the suggested upper and lower values of the variables that require optimization as they establish the starting point of the decision variables. The steps used to identify the best solutions to the optimization problem are provided below.

#### Initialization

The GA aimed to solve the optimization problem by searching for an optimal solution; which was the lowest cost; among the variables of the problem; which were the P_WIND_, P_PV_, P_BAT1_, P_BAT3_, P_BAT2_, and P_GRID_. Thus, the first step of the GA was fitting by specifying an individual as an array of variable values to be optimized. In this present study, six variables (Nvar = 6) were associated with the individual, known as the chromosome.

18 $${\text{Chromosome}}=[{{\text{P}}}_{{\text{PV}}}{{\text{P}}}_{{\text{WIND}}}{{\text{P}}}_{\mathrm{Bat }1}{{\text{P}}}_{\mathrm{Bat }2}{{\text{P}}}_{\mathrm{Bat }3}{{\text{P}}}_{{\text{GRID}}}]$$

The GA can function with various possible solutions, known as populations. A population can include 30 to 100 chromosomes. A matrix denotes the population within each row of the matrix, which is a 1 × Nvar chromosome of the continuous values. By considering a 24-chromosome preliminary population of Npop, the matrix below describes the full Npop × Nvar matrix of the random values:

$$\left[\begin{array}{cccccc}{\text{PPV}}1& {\text{PWIND}}1& {\text{PBAT}}1,1& {\text{PBAT}}2,1 & {\text{PBAT}}3,1& {\text{PGRID}}1\\ {\text{PPV}}2& {\text{PWIND}}2& {\text{PBAT}}1,2& {\text{PBAT}}2,2& {\text{PBAT}}3,2& {\text{PGRID}}2\\ {\text{PPV}}3& \mathrm{ PWIND}3& {\text{PBAT}}1,3& {\text{PBAT}}2,3& {\text{PBAT}}3,3& {\text{PGRID}}3\\ \dots & \dots & \dots & \dots & \dots & \dots \\ \dots & \dots & \dots & \dots & \dots & \dots \\ {\text{PPV}}24 & {\text{PWIND}}24& {\text{PBAT}}1,24& {\text{PBAT}}2,24& {\text{PBAT}}3,24& {\text{PGRID}}24\end{array}\right]$$

#### Selection and crossover

A selection procedure helps determine which parents need to die and which ones must be preserved and allowed to reproduce to maintain a constant population size. Meanwhile, a crossover is the basic operator used to generate new chromosomes in the proposed algorithm. Much like in nature, a crossover can generate new chromosomes that include a few sections of the genetic materials of both parents. A crossover was employed to yield new solutions based on the available existing solutions in the mating group post-use of the selection operator.

#### Mutation

**Figure 10:**
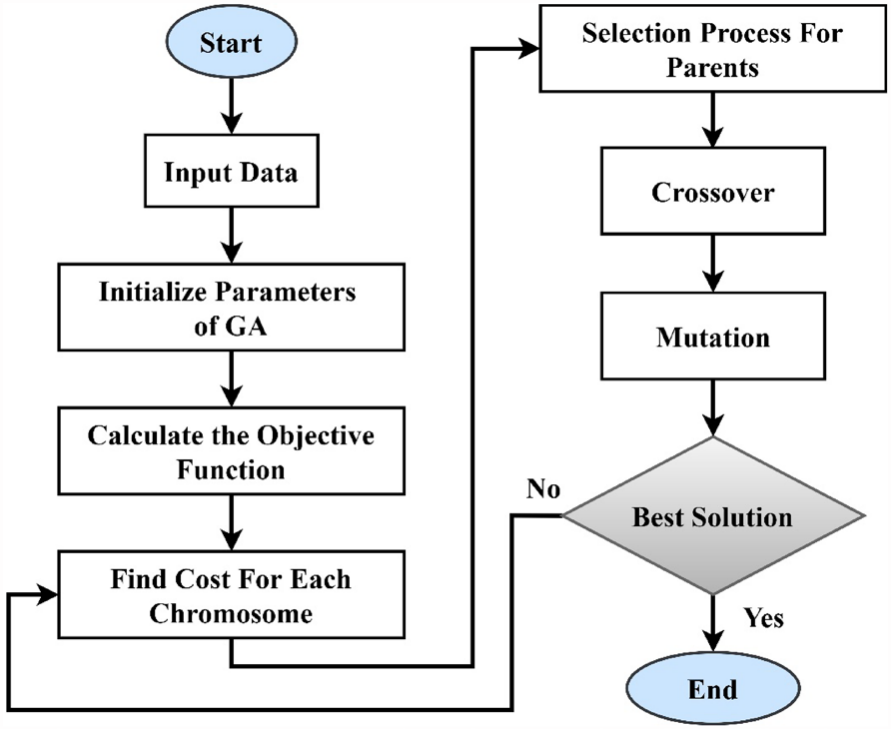
The GA stages flowchart.

A mutation can be defined as a random process that is responsible for searching for the best and most optimal solution. The mutation process begins with selecting the mutation rate and the variables’ row while the columns to be mutated are selected based on random numbers. A new random variable then switches a mutated variable based on the variable's limits. The stages of the GA are presented in the flowchart provided in Fig. 10.

## Simulation results and discussions

This present study used RB-EMS, GA and SAA. In the testing phase, the RB-EMS considered the behaviour during the day, the PV-related irradiation data at a constant temperature of 25 °C, the random demand profile, and the wind data speed for one day. The RB-EMS was tested based on the grid that was connected to the NG, which comprised a WT system, a PV array and three BSDs. Both the RESs and BSDs had to supply the load at any given time to ensure constant power flow. The GA optimization and its effect on the overall cost of the NG system was presented. The cost saving efficacy of the RB-EMS was also compared with GA and SAA.

**Figure 11:**
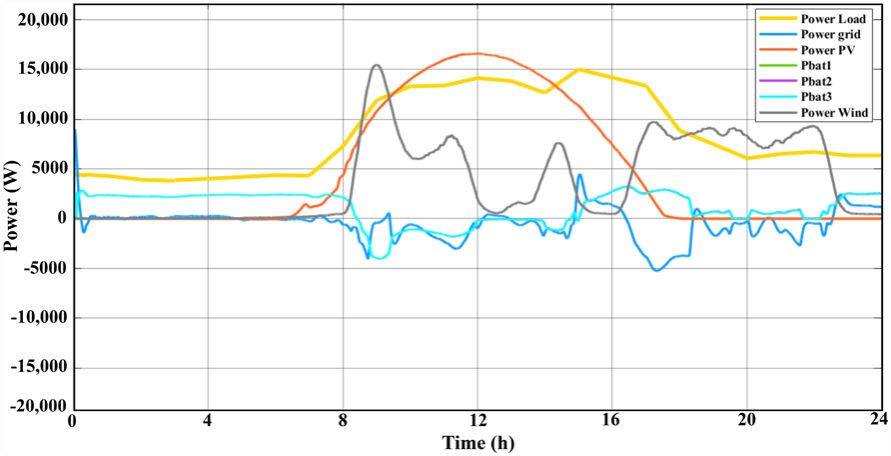
PV, wind, batteries, load, and grid power curves for 1 day.

**Figure 12:**
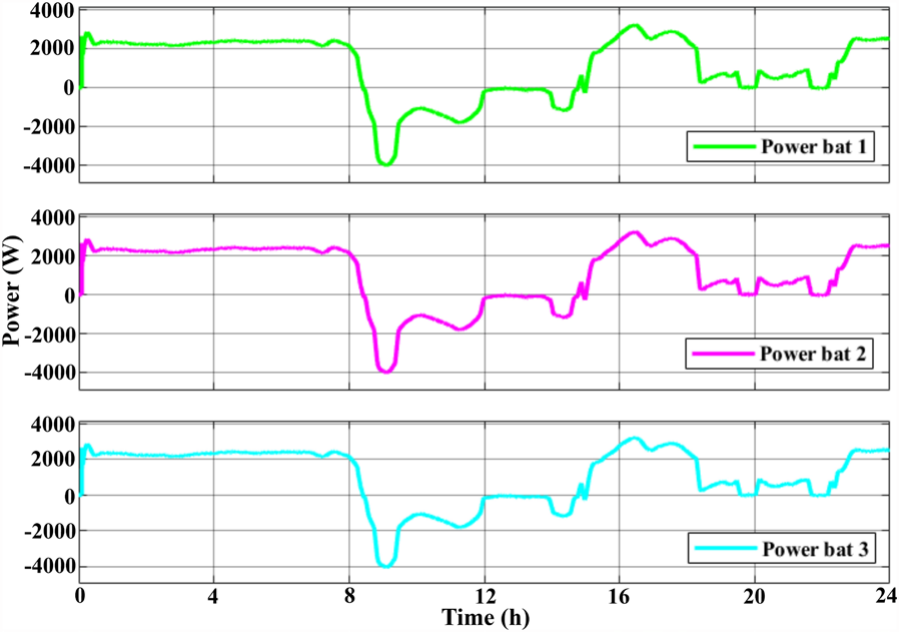
The batteries charge and discharge curves for 1 day.

The purpose of the RB-EMS was to satisfy load demands that had reached a peak of 15 KW in various generating conditions and utilising minimum supply from the grid. Figure 11 shows the power at different locations of the NG. The three BSDs supplied the load for up to 8 h as the PV and WT produced zero power. Post 8 h, the PV and WT supplied the load at various intervals while the additional power produced was used to charge the BSD. The excess power was then transferred to the grid for up to 15 h. The BSD supplied the load as the both the PV and WT produced less power between 15 and 18 h. Between 18 and 24 h, the WT supplied the load with support from the BSD. Figure 12 shows the discharging and charging states of the three BSDs during the day while Table 6 lists the total power produced by the WT and PV, the discharging and charging states of the BSD, and the power transferred to the grid over a 24 h period post-using the RB-EMS.

**Table 6:** The power of each source after applying RB-EMS.

Time (h)	P_PV (kW)	P_WIND (kW)	P_BAT1 (kW)	P_BAT2 (kW)	P_BAT3 (kW)	P_GRID (kW)
1	0	0	2.3758	2.3758	2.3758	0.026701
2	0	0	2.2223	2.2223	2.2223	0.19228
3	0	0	2.2025	2.2025	2.2025	0.18756
4	0	0	2.3831	2.3831	2.3831	0.23742
5	0	0	2.3611	2.3611	2.3611	− 0.10344
6	0	0.13385	2.3973	2.3973	2.3973	− 0.064919
7	1.2225	0.21054	2.2913	2.2913	2.2913	− 0.26211
8	5.1682	0.50225	1.7427	1.7427	1.7427	− 0.33055
9	10.771	15.484	− 3.9865	− 3.9865	− 3.9865	− 0.4598
10	13.988	6.2973	− 1.0701	− 1.0701	− 1.0701	− 0.58628
11	15.902	7.632	− 1.5901	− 1.5901	− 1.5901	− 2.3206
12	16.604	1.9984	− 0.18209	− 0.18209	− 0.18209	− 1.0174
13	15.928	1.3477	− 0.10251	− 0.10251	− 0.10251	− 0.16861
14	14.1	4.7813	− 0.72559	− 0.72559	− 0.72559	− 1.3571
15	11.314	1.8135	− 0.0023817	− 0.0023817	− 0.0023817	4.407
16	7.4909	0.49032	2.7134	2.7134	2.7134	1.1682
17	2.9568	8.8299	2.5064	2.5064	2.5064	− 3.7322
18	0.017231	8.1933	2.4362	2.4362	2.4362	− 3.8084
19	0	9.0514	0.70303	0.70303	0.70303	− 1.5764
20	0	8.3478	0.018947	0.018947	0.018947	− 0.2858
21	0	7.6961	0.63583	0.63583	0.63583	− 1.3901
22	0	9.2805	− 0.0070803	− 0.0070803	− 0.0070803	− 0.53016
23	0	0	2.5112	2.5112	2.5112	1.61
24	0	0	2.5604	2.5604	2.5604	1.1341

As seen in Fig. 11, the power outputted by all the sources post-using the RB-EMS was extracted to the workspace and acted as an input for the GA. The optimization problem was solved based on the optimization algorithm, which helped decrease the overall cost of the NG. The daily PV irradiation data and the wind speed data were used to analyse the results. The SOC of the BSD constraints were kept in the range of 20 to 80% SOC, which helps extend battery life. The NG used a lithium-ion battery. As for the GA optimization operation, the time step was set to a 24 h scale. The GA optimization helped decrease the COE from the main grid as well as satisfy the load demand using the power generated by the WT, the PV, the grid, and the BSDs, which, in turn, helped decrease the COE of the grid. Moreover, a comparison with SAA has been presented to showcase the effectiveness of the GA. Table 7 lists the optimum allocation post-using GA for NG generators, BSDs, and the main grid in a 24 h operation scenario, as per the cost function. While Table 8 presents the list of ideal distributions after the use of SAA for the NG generators, BSDs, and the primary grid in the case of a 24 h operation context, based on the cost functions.

**Table 7:** The power of each source after applying GA.

Time (h)	P_PV (kW)	P_WIND (kW)	P_BAT1 (kW)	P_BAT2 (kW)	P_BAT3 (kW)	P_GRID (kW)
1	0	0	2.3755	2.3756	2.3756	− 2.8362
2	0	0	1.4892	1.487	1.4876	− 0.545
3	0	0	1.4683	1.4686	1.4689	− 0.54673
4	0	0	2.3831	2.3831	2.3821	− 3.1423
5	0	0	2.3593	1.4901	1.3039	− 0.97427
6	0	0.13023	2.3963	2.3973	2.3973	− 2.8279
7	1.2217	0.17252	2.2912	2.2913	2.2913	− 3.7825
8	4.8294	0.20242	1.2833	1.7424	1.2772	− 1.8803
9	0.16613	14.33	− 3.9865	− 3.9865	− 3.9865	− 2.6169
10	11.846	4.1736	− 3.0036	− 1.3087	− 1.1066	− 2.7211
11	15.902	7.6318	− 1.6148	− 1.6176	− 1.6103	− 10.174
12	13.138	1.9984	− 0.19813	− 0.19113	− 3.64	− 1.0185
13	15.928	1.3471	− 1.3969	− 2.1735	− 3.9017	− 3.4506
14	13.141	2.8238	− 1.7352	− 1.2622	− 0.77025	− 3.3162
15	10.138	1.636	− 3.9364	− 2.9847	− 0.052179	3.225
16	7.0509	0.49025	1.834	2.7202	2.2806	0.28731
17	2.5045	8.6287	2.4963	2.3668	2.499	− 5.1482
18	0	7.477	2.4362	2.4448	1.7287	− 5.2426
19	0	9.0514	0.70303	0.69151	0.69151	− 1.5764
20	0	8.3473	0.018947	− 0.013587	− 0.013587	− 2.3032
21	0	7.6961	0.63583	0.61402	0.61402	− 1.3901
22	0	9.2796	− 0.0087762	− 0.85369	− 0.017254	− 2.5806
23	0	0	2.0558	2.0601	2.0629	0.15312
24	0	0	2.4514	1.7367	1.9796	0.15312

**Table 8:** The power of each source after applying SSA.

Time (h)	P_PV (kW)	P_WIND (kW)	P_BAT1 (kW)	P_BAT2 (kW)	P_BAT3 (kW)	P_GRID (kW)
1	0	0.0024	1.4706	1.2967	1.6039	− 0.0825
2	0	0.0037	1.0081	1.2272	1.7025	− 0.022
3	0	0.0025	1.0798	1.2983	1.3219	0.1573
4	0	0.0136	0.9445	1.483	1.1259	0.4396
5	0	0.0373	1.0007	1.3068	1.6078	0.2256
6	0	0.0183	1.4370	1.6282	1.5106	− 0.2302
7	0.4005	0.2101	0.7925	1.7132	0.9723	0.2251
8	3.0205	0.4011	1.4586	0.9704	1.5059	− 0.1038
9	9.4301	13.4799	− 3.9864	− 3.9864	− 3.9864	− 0.4063
10	12.3936	5.4318	− 1.4786	− 1.3434	− 1.2694	− 0.4339
11	14.3671	6.4831	− 1.8233	− 1.7111	− 2.2557	− 1.7001
12	14.0401	1.6545	− 0.4847	− 0.2702	− 0.4054	− 0.4152
13	13.6151	1.1518	− 0.3674	− 0.1163	− 0.3833	− 0.0747
14	12.1775	3.9105	− 0.729	− 0.7773	− 0.7578	− 1.1737
15	10.8265	1.4049	− 0.2896	− 0.0599	− 0.4653	3.5834
16	6.1777	0.3896	2.2472	2.0062	2.386	0.9671
17	2.7211	8.3008	2.1359	2.3943	1.9599	− 4.1642
18	0	6.357	1.6767	2.2382	1.5252	− 2.9522
19	0	7.8848	0.4429	0.3518	0.2802	− 1.4598
20	0	6.8184	− 0.136	− 0.3759	− 0.1208	− 0.1405
21	0	6.0188	0.4813	0.5396	0.5921	− 1.132
22	0	8.0904	− 0.4505	− 0.4872	− 0.4545	0.0018
23	0	0.6535	1.5094	1.6575	1.6873	0.8239
24	0	0.0827	1.6193	1.7706	1.7304	1.1298

**Figure 13:**
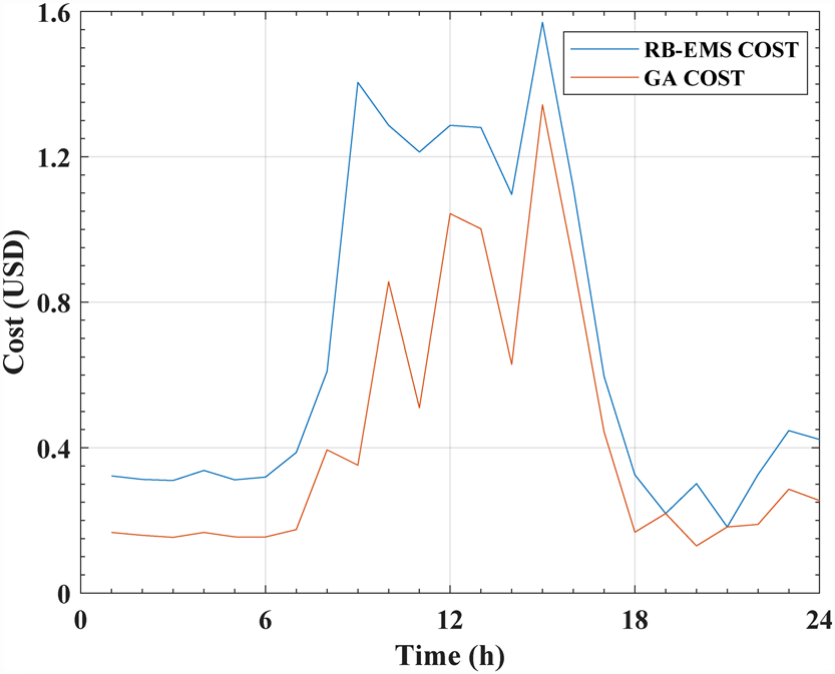
The cost of RB-EMS and GA for NG system.

**Figure 14:**
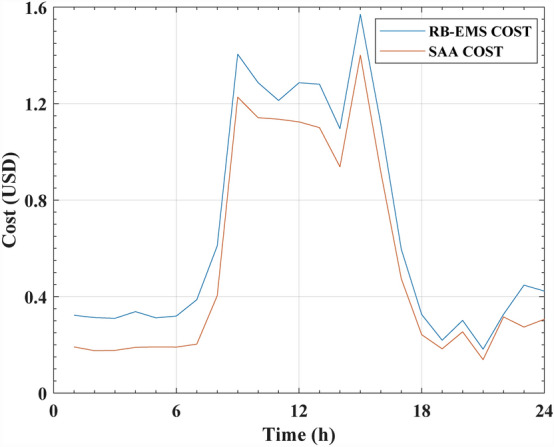
The cost of RB-EMS and SAA for NG system.

The GA optimization provided an added benefit for almost every measure versus the RB-EMS. The total cost of variable-price electricity was calculated in terms of USD/kWh. The results showed that the COE of RB-EMS was USD 15.9856/day whereas the COEs displayed by the GA and SAA optimization systems were USD 9.50672/day and USD 12.8928/day, respectively. Table 9 presents the COE (per hour) and the daily overall cost savings after employing the RB-EMS and GA systems. Figure 13 displays the COE values shown by the GA and RB-EMS systems. Table 10 displays the COE (per hour) and the daily overall cost savings after employing the RB-EMS and SAA systems; whereas Fig. 14 depicts the COE comparison of both the systems.

**Table 9:** RB-EMS and GA cost and the saving for one day.

Time (h)	Cost after applying RB-EMS (USD)	Cost after applying GA (USD)	Saving (USD)
1	0.32252	0.13067	0.19185
2	0.3129	0.16435	0.14854
3	0.30991	0.16163	0.14828
4	0.33763	0.11114	0.22649
5	0.31181	0.16662	0.14519
6	0.31929	0.13412	0.18516
7	0.38712	0.15119	0.23593
8	0.61029	0.41049	0.1998
9	1.4049	0.34938	1.0555
10	1.2869	0.8555	0.43136
11	1.2131	0.11355	1.0996
12	1.2866	1.0161	0.27047
13	1.2806	1.0016	0.279
14	1.0963	0.67089	0.42542
15	1.5702	1.3061	0.26415
16	1.1148	0.89776	0.21701
17	0.59643	0.42557	0.17086
18	0.32549	0.14342	0.18207
19	0.21901	0.21901	0
20	0.30127	0.12977	0.1715
21	0.18199	0.18199	0
22	0.32642	0.18901	0.13742
23	0.4473	0.28831	0.15899
24	0.42289	0.28855	0.13434
Total	15.9856 USD/day	9.50672 USD/day	6.47892 USD/day

**Table 10:** RB-EMS and SAA cost and the saving for one day.

Time (h)	Cost after applying RB-EMS (USD)	Cost after applying SAA (USD)	Saving (USD)
1	0.32252	0.19118	0.13133
2	0.3129	0.17572	0.13716
3	0.30991	0.17704	0.13286
4	0.33763	0.18937	0.14825
5	0.31181	0.19131	0.12049
6	0.31929	0.19048	0.12879
7	0.38712	0.20284	0.18427
8	0.61029	0.40385	0.20643
9	1.4049	1.22673	0.17817
10	1.2869	1.14166	0.14519
11	1.2131	1.13546	0.07765
12	1.2866	1.12436	0.16224
13	1.2806	1.10054	0.18003
14	1.0963	0.93803	0.15827
15	1.5702	1.40093	0.16927
16	1.1148	0.91604	0.19872
17	0.59643	0.47408	0.12234
18	0.32549	0.24179	0.08368
19	0.21901	0.18342	0.03559
20	0.30127	0.25397	0.04729
21	0.18199	0.13851	0.04348
22	0.32642	0.31565	0.01076
23	0.4473	0.27364	0.17365
24	0.42289	0.30611	0.11677
Total	15.9856 USD/day	12.8928 USD/day	3.0928 USD/day

The results presented in Figs. 13 and 14 indicated that with regard to the determination of optimal operating costs, the proposed GA showed a better performance compared to the SAA algorithm. It was noted that the GA acquired a 40% cost saving, while the SAA achieved a 19.3% cost saving compared to the RB-EMS. The primary difference between the optimization algorithms and RB-EMS was that the RB-EMS relied mainly on logic and predefined rules before making decisions regarding the distribution and consumption of energy in the NG systems. These rules were based on the system’s knowledge and operational experience. This technique was generally used for guaranteeing power flow in the NG system and for providing safe and reliable functioning.

Meanwhile, optimization algorithms like GA employ mathematical models for determining the cost-effective and efficient ways of managing energy in the NG system. The above algorithms consider different constraints and factors for optimizing energy generation, storage, and energy consumption, for fulfilling objectives like cost minimization.

The benefits of the GA optimization for the NG system can be described as follows: The GA significantly decreases the COE of NG compared to RB-EMS. Moreover, the proposed optimization algorithm also reduced grid energy usage. In future, several advanced algorithms will be used for solving the multi-objective issue that includes minimizing the costs and emissions generated from the NG system.

## Conclusions

In this study, an RB-EMS is proposed for the grid-connected NG that included a PV, WT, and BSD. The RB-EMS primarily aimed to offer safe and reliable functioning and guarantee a steady power flow within the NG system. Furthermore, the GA was employed for minimizing the costs of the NG system. In addition to its simplicity, the GA-based optimization technique showed a low computational complexity. The GA technique proposed in this study could effectively overcome the limitations in comparison to the conventional heuristic techniques. This study compared the simulation results presented by the GA, RB-EMS, and SAA optimization techniques; wherein the daily costs for the RB-EMS, SAA, and GA techniques were seen to be USD 15.9856/day, USD 12.8928/day, and USD 9.50672/day respectively. Finally, it was concluded that the GA-based optimization technique was very cost-effective. This technique also lowered the variable-electricity cost by ≈ 40% compared to the RB-EMS and also lowered the grid energy usage. On the other hand, the SAA optimization reduced the costs by ≈ 19.3% compared to the RB-EMS.

## Data Availability

The datasets used and/or analyzed during the current study are available from the corresponding author on reasonable request.
